# Antimicrobial resistance and molecular characteristics of *Neisseria gonorrhoea* isolates in Ghana

**DOI:** 10.1099/acmi.0.000689.v3

**Published:** 2024-02-08

**Authors:** Haris Sualah Musah, Francis Addy, Osman Adamu Dufailu

**Affiliations:** ^1^​ Department of Biochemistry and Molecular Medicine, School of Medicine, University for Development Studies, Box 1350, Tamale, Ghana; ^2^​ Department of Biotechnology, Faculty of Biosciences, University for Development Studies, Box 1882, Tamale, Ghana; ^3^​ Department of Microbiology, Faculty of Biosciences, University for Development Studies, Box 1882, Tamale, Ghana; ^4^​ School of Science, University of Greenwich, Central Avenue, Kent ME4 4TB, UK

**Keywords:** *N. gonorrhoea*, antimicrobial resistance, molecular characterization, Ghana

## Abstract

**Introduction.:**

Gonorrhoea is a disease associated with humans and caused by *Neisseria gonorrhoea. N. gonorrhoea’*s ability to evolve and evade various treatment regimens can lead to untreatable gonorrhoea. In the absence of a viable vaccine and a national database on the antimicrobial resistance (AMR) and molecular characteristics of *N. gonorrhoea,* and with reliance on a syndromic management regime, continuous national antimicrobial resistance surveillance and molecular characterization of *N. gonorrhoea* remain imperative. Only two gonococcal studies have described *N. gonorrhoea’s* molecular characteristics linked to AMR in Ghana.

**Methods.:**

Secondary *N. gonorrhoea* isolates (*n*=4) were collected from two metropolises in Ghana: Tamale in the northern sector (*n*=1) and Accra in the southern sector (*n*=3). The isolates were confirmed and characterized using polymerase chain reaction (PCR) targeting the *por*B and *tbp*B genes, and the disc diffusion method was used to evaluate AMR. *N. gonorrhoea* multi-antigen sequence typing (NG-MAST) and porin B (*por*B) gene sequence analyses were employed to reveal the molecular epidemiology and evolutionary trajectory, respectively.

**Results.:**

All four isolates showed resistance to at least four of the tested antibiotics. One isolate showed resistance to all seven antibiotics, i.e. ceftriaxone, azithromycin, ciprofloxacin, tetracycline, erythromycin, togamycin and penicillin. NG-MAST typing revealed isolate S3 (MZ313864) as ST211. The locus of S2 (MZ313863) (transferrin-binding protein B; *tbp*B) was identified as *tbp*B1844, and its *por*B locus, as *por*B6412, with only 4 closely related variants but with 15 nucleotide differences. However, its sequence type does not exist. The *por*B analysis identified isolate S3 (MZ313864) to be found globally, while S2 (MZ313863) is unique to this study.

**Discussion.:**

Despite the small number of isolates tested, this study recorded multidrug resistance and previously unknown gonococcal variants based on *por*B gene. Additionally, the molecular typing schemes revealed a disparity between NG-MAST and the National Center for Biotechnology Information (NCBI) platforms. There is a need for continuous gonococcal AMR and molecular surveillance in Ghana to contribute to the global efforts to describe circulating strains and support proper application of the syndromic management regime to gonorrhoea.

## Data Availability

Sequencing data have been deposited in the National Center for Biotechnology Information’s (NCBI’s) GenBank with the following accession numbers: MZ313863 and MZ313864.

## Introduction

*Neisseria gonorrhoea* is a human-associated pathogen that causes gonorrhoea. The World Health Organization (WHO) in 2020 estimated 82 million global incidences of gonorrhoea among young adults [1].

Gonorrhoea is associated with infections such as urethritis, cervicitis, arthritis, endocarditis, infertility and meningitis, among other deleterious conditions, when treatment is delayed or unsuccessful [2, 3]. Other complications, such as inflammation of the foetal membrane, infectious miscarriage, premature birth and early rupture of the amniotic sac, have been reported in pregnant women with gonorrhoea infection [4]. During vaginal delivery, gonorrhoea is reported to cause blindness in newborns via vertical transmission [2, 3].

Prior to antimicrobials, several treatments of gonorrhoea included crude and painful techniques such as genital hyperthermia [5]. However, the use of antimicrobials in the treatment of gonorrhoea has been successful in the absence of a viable vaccine until the emergence of multidrug-resistant strains of *N. gonorrhoea* [5, 6]. This development has made multidrug-resistant *N. gonorrhoea* a global health concern [5].

The ability of *N. gonorrhoea* to resist antimicrobials differs in various parts of the world and at different times [7–10]. The lack of effective diagnostic tools and the over-reliance on symptomatic diagnosis and treatment in lower-income countries, especially in Africa, may contribute to the widespread emergence of resistant gonococcal strains [5, 9].

NG-MAST, a widely used epidemiological online platform currently hosted by www.pubmlst.org as ng-mast v2.0 [11], has been employed in identifying clusters of gonococcal strains with the same characteristics [12]. Furthermore, *por*B gene sequence analysis has generally been applied in the study of the evolutionary trajectory, either alone or with other genes of the gonococcus [13, 14]. Thus, global efforts to better appreciate the antimicrobial resistance (AMR) profile and genetic diversities of circulating *N. gonorrhoea* strains remain critical. This is useful for describing circulating gonococcal strains and informing the application of local syndromic treatment regimen.

In Ghana, and as a snapshot of the African picture, only two studies [15] and [16] have reported on the molecular characteristics of *N. gonorrhoea*. There are a few individual publications [15, 17] on AMR, but there is no recognized national AMR database of gonococcal isolates to inform the adopted symptomatic treatment regime. However, Ghana’s Ministry of Health has continuously updated the syndromic management regime of gonorrhoea using WHO recommendations [18]. Extensive descriptions of locally circulating gonococcal strains can help improve the syndromic management practice for gonorrhoea in Ghana. Such studies will also help understand the multiple antibiotic resistance (MAR) index, which describes the exposure of specific antibiotics to organisms and to which high resistance or susceptibility is observed. A MAR index above 0.2 indicates the extensive or high-risk use of an antibiotic in a setting [19]. To describe *N. gonorrhoea* in the country and promote an informed localized symptomatic management regimen, the current study evaluated the antimicrobial resistance and molecular characteristics of gonococcal isolates in Ghana.

## Methods

### Gonococcal isolates

Secondary gonococcal isolates from the Tamale Teaching Hospital (TTH) (*n*=1), Korle-Bu Teaching Hospital, Accra (*n*=2), and MDS Lancet Laboratories, Accra (*n*=1) were employed in this study. The TTH isolate was from an antenatal surveillance study involving 230 swab samples (Musah *et al*., unpublished data), while the Accra isolates were from routine clinical diagnoses of gonorrhoea at the stated facilities. Collected suspected gonococcal isolates from Accra were cryopreserved in 85 % peptone–15 % glycerol solution and transported in an ice pack to Tamale. All collected isolates were stored at −20 °C in the peptone–glycerol solution until required for laboratory analysis. Gonococcal reference strain ATCC 49226, received from Noguchi Memorial Research Institute, Ghana, was included as a positive control.

### Media preparation

GC agar (Oxoid, UK) supplemented with Vitox (Oxoid, UK) and 5 % sheep blood were prepared following the manufacturer’s instructions. Briefly, 18 grams of GC agar base was weighed and dissolved in 500 ml of distilled water. The mixture was then sterilized in an autoclave for 15 min at 121 °C and allowed to cool to ~50 °C. Defibrinated sheep blood and Vitox supplement prepared following the manufacturer’s instructions were used to supplement the media.

### Culturing of *N. gonorrhoea* isolates

Collected *N. gonorrhoea* isolates were inoculated on the GC agar supplemented with Vitox and incubated at 37 °C with moistened cotton in a candle jar for 24–48 h as previously described [20].

### PCR confirmation and amplification of *por*B and *tbp*B genes of *N. gonorrhoea*

*N. gonorrhoea* isolates were confirmed by the PCR amplification of two conserved genes, *por*B (737 bp) and *tbp*B (589 bp), as previously described [12], with slight modifications. Briefly, crude DNA was obtained by suspending 8 to 10 colonies in 30 µl Tris buffer (pH 7.3) and heated at 100 °C for 10 min. A 50 µl final reaction PCR mix of either *por*B or *tbp*B developed in the laboratory comprised 5 µl crude DNA, 25 µl of One*Taq* 2× Master Mix (New England BioLabs, Inc., UK), and 6.25 µl each of forward and reverse primers (10 µM) (synthesized by Inqaba Biotec Ltd, South Africa) of either *por*B or *tbp*B genes, while 7.5 µl of nuclease-free water was added to make up the 50 µl final volume. Gonococcal reference strain ATCC 49226 was used as a positive control, while nuclease-free water was used as a negative control in place of DNA. The PCR cycle conditions, as previously described [12], were carried out on a peqSTAR 96 Universal Gradient thermal cycler (VWR-USA). For the *tbp*B gene, initial denaturation was at 95 °C for 4 min, followed by 25 cycles of 95 °C for 30 s denaturation, 69 °C for 30 s for annealing and 72 °C for 1 min for the extension. The final extension was for 10 min before cooling and stored at 4 °C. All the PCR conditions for the *por*B gene were the same as the *tbp*B gene except for the annealing condition, which was 58 °C for 30 s.

Nine microlitres of PCR product for each gene mixed with 1 µl 6× gel loading dye (Thermo Fisher Scientific, USA) was resolved on a 1.4 % agarose gel for 50 min at 80 V. A 100 base DNA ladder (FastRuler, Thermo Fisher Scientific, USA) was used as a DNA marker. A UV trans-illuminator (Cleaver Scientific Ltd, UK) with a mounted Canon Power Shot G16 camera (Canon, Inc., Japan) was used to visualize and document the gels.

### AMR of confirmed *N. gonorrhoea*

The resistance profile of the confirmed *N. gonorrhoea* for seven antibiotics (Mast Group Ltd, UK) – penicillin (PG: 10 units), azithromycin (ATH: 15 µg), ceftriaxone (CRO: 30 µg), ciprofloxacin (CIP: 5 µg), erythromycin (E: 15 µg), togamycin (TG: 10 µg) and tetracycline (T: 30 µg) – was evaluated using the disc diffusion method following the procedure and guidelines of the Clinical and Laboratory Standards Institute (CLSI) [21]. For azithromycin, the Centers for Disease Control and Prevention (CDC’s) breakpoint was used to interpret its results. Triplicate averages of the inhibition zones were used for this purpose. The ratio of antibiotics resisted by an isolate to the number of antibiotics tested on the isolate, multiplied by 100%, was used to indicate an antibiotic’s antimicrobial resistance percentage. Multiple AMR index was evaluated as a ratio of the number of antibiotics an isolate is resistant to the number of antibiotics evaluated on the isolate as previously described [19].

### Sequencing of amplified gonococcal genes

The *por*B and *tbp*B gene PCR amplicons of the confirmed gonococcal isolates and reference strain ATCC 49226 were sequenced by Inqaba Biotec. Ltd, South Africa using Sanger sequencing technology on an ABI Genetic Analyser 3500XL (Thermo Fisher Scientific, UK). DNA sequence chromatograms were viewed, trimmed and edited using GENtle (v1.8.0) [22] and Molecular Evolution Genetics Analysis X (mega X) [23] software.

### NG-MAST analysis

NG-MAST analysis was carried out as described previously [12]. Briefly, gonococcal DNA sequences were trimmed at consensus regions starting from nucleotide (nt) 445 in reference to *N. gonorrhoea* strain MS11 to yield 490 base pairs (bp) for *por*B and for *tbp*B, from nt 1118 reference to strain UU1008 to yield 390 bp. These were submitted to the NG-MAST portal, currently held at www.pubmlst.org as ng-mast v2.0 [11] to search against the database’s sequence repository as previously described. Each sequence (*por*B or *tbp*B) of the same isolate describes a locus type, while the combination of the loci describes a sequence type (ST).

### *N. gonorrhoea por*B sequence analysis

The *por*B gene sequence lengths were trimmed to yield a maximum of 510 bp based on the readability of the sequence chromatograms [24] starting from nucleotide 1 990 471 to 1 990 980 relative to *N. gonorrhoea* strain WHO N genome assembly, chromosome 1 (accession no.: LT591910.1). The National Center for Biotechnology Information’s (NCBI) online Basic Local Alignment Search Tool (blast) [25] was used to compare sequence similarities with other *N. gonorrhoea* sequence deposits and to identify their isoforms. Default blast settings and the ‘highly similar sequences’ algorithm, megablast, were used for the search. Subsequently, intra-sequence variation was determined using default ClustalW [26] pairwise alignment algorithm settings on GENtle software v.1.9.4 http://gentle.magnusmanske.de [22] (University of Cologne). A global haplotype analysis network was constructed on Population Analysis with the Reticulate Tree (PopArt v.1.7; http://popart.otago.ac.nz) [27] software using the theoretical computer science (TCS) algorithm to visualize the genealogical relatedness of the gonococcal isolates with 23 selected haplotypes from the blast hit result. The haplotypes were selected randomly to cover countries in the selected maximum target sequences of 100 hits of the blast results. Molecular Evolution Genetics Analysis (mega) version X software was employed to align the two isolates in this study and the randomly selected 23 blast haplotypes using the default settings of ClustalW and maximum-composite-likelihood model to draw the phylogenetic tree while employing 1000 bootstrap replications to test the confidence of the tree.

## Results

### PCR confirmation of presumptive *N. gonorrhoea* isolates

**Fig. 1.**
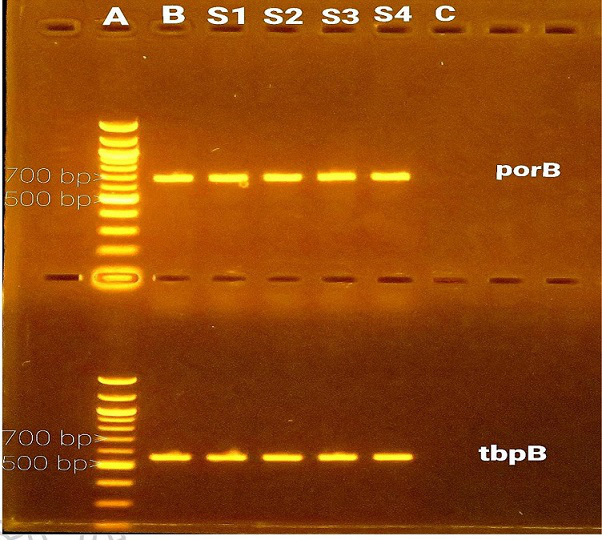
PCR confirmation of gonococcal isolates S1, S2, S3 and S4 showing the *por*B (737 bp) top and *tbp*B (589 bp) bottom amplifications: (a) 100 bp DNA markers, (b) *N. gonorrhoea* reference strain (ATCC 49226) as positive control and (c) negative control.

PCR confirmed the collected gonococcal (secondary isolates) before AMR studies were carried out. All four isolates were confirmed as *N. gonorrhoea* using *por*B and *tbp*B genes. The PCR of the confirmed gonococcal isolate from Tamale was assigned S2, while the other three confirmed isolates from Accra were assigned S1, S3 and S4 (See Fig. 1).

### Antimicrobial resistance of confirmed *N. gonorrhoea* isolates

**Fig. 2.**
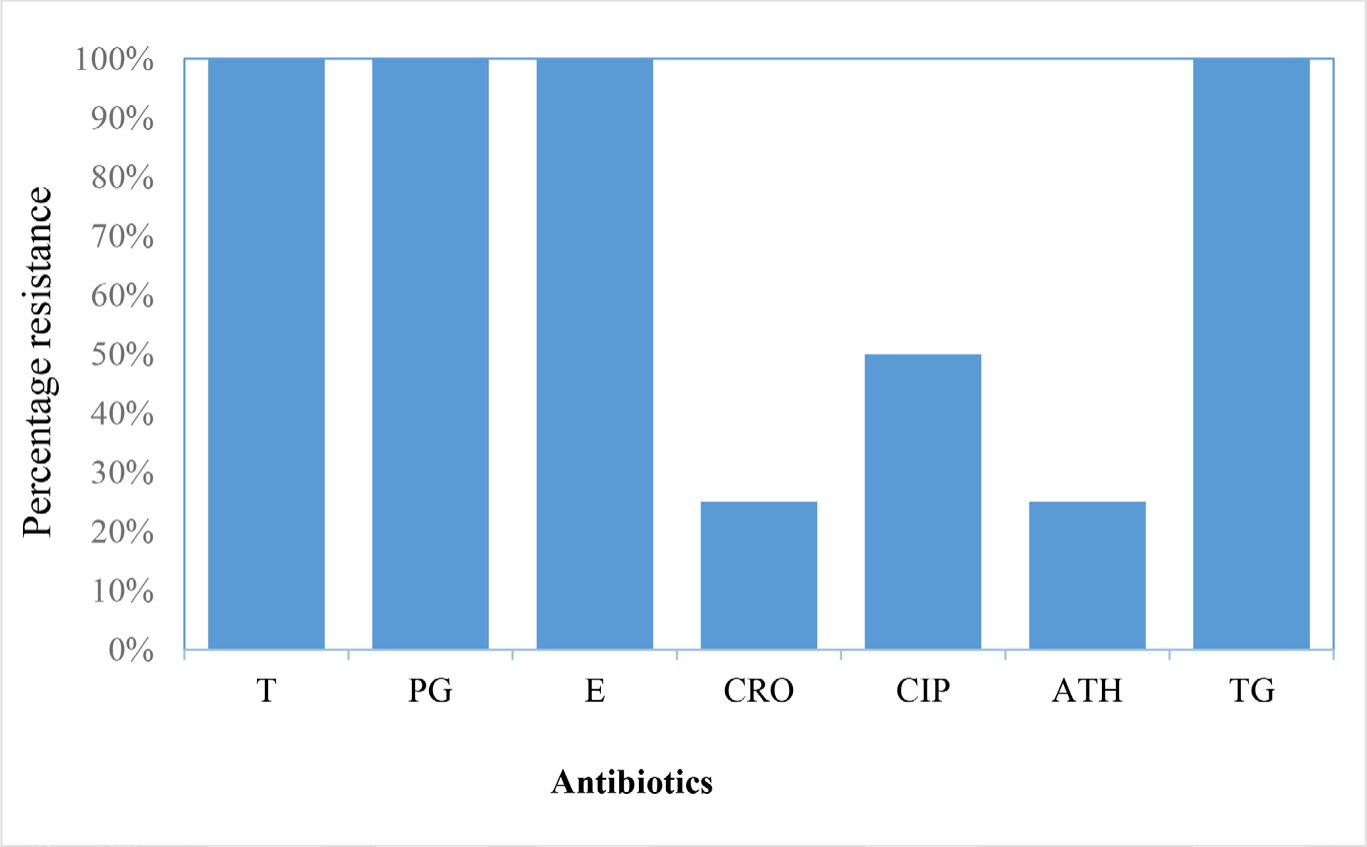
Percentage resistance to seven antibiotics for four *N. gonorrhoea* isolates. T, tetracycline; PG, penicillin; E, erythromycin; CRO, ceftriaxone; CIP, ciprofloxacin; ATH, azithromycin; TG, togamycin.

Following the evaluation of the AMR of the confirmed *N. gonorrhoea* isolates using the CLSI guidelines and breakpoints, all four gonococcal isolates exhibited resistance or intermediate susceptibility to all seven antibiotics tested except ceftriaxone. Ceftriaxone resistance was observed in isolate S4. Furthermore, all four isolates revealed a multiple antibiotic resistance index >0.5. Table 1 shows the gonococcal isolates' AMR profile against the seven tested antibiotics. Fig. 2 illustrates the percentage resistance of the four gonococcal isolates to the seven antimicrobials.

**Table 1.** Antimicrobial resistance profile of four *N. gonorrhoea* isolates against CLSI and CDC’s breakpoints interpretation of seven antibiotics. R, S, and I represent resistance, susceptibility and intermediate susceptibility, respectively. Whereas T- Tetracycline, PG- Penicillin, E- Erythromycin, CRO- Ceftriaxone, CIP-Ciprofloxacin, ATH-Azithromycin, TG-Togamycin. MAR denotes multiple antibiotic resistance index, which is a ratio of resistant antibiotics to the total number of antibiotics evaluated

Antibiotics (*n*=7)	S1	S2	S3	S4
T (30 µg)	R	R	R	R
PG (10 U)	R	R	R	R
E (15 µg)	R	R	R	R
CRO (30 µg)	S	S	S	R
CIP (5 µg)	R	I	I	R
ATH (15 µg)	I	I	I	R
TG (10 µg)	R	R	R	R
**MAR index**	**0.71**	**0.57**	**0.57**	**1.00**

ATH, Azithromycin ; CIP, Ciprofloxacin ; CRO, Ceftriaxone ; E, Erythromycin ; I, Intermediate susceptibility,; MAR, Multiple antibiotic resistance; PG, Penicillin ; R, Resistance; S, Susceptibility; T, Tetracycline ; TG, Togamycin .

### Molecular characterization of *N. gonorrhoea* using *por*B and *tbp*B genes

Following two DNA sequencing attempts of the four gonococcal isolates and the reference strain, only the sequence chromatograms of S2 and S3 were readable for both genes (i.e. *por*B and *tbp*B). Therefore, sequences of S2 and S3 were trimmed appropriately for downstream molecular characterization.

### Molecular typing (NG-MAST) of *N. gonorrhoea*

Isolate S3 was identified as NG-MAST ST 211 (ST211). A locus search of the S2 *tbp*B gene identified the allele as *tbp*B1844 with three STs (ST10251, ST19711 and ST19719). The *por*B gene of S2 was identified closest to *por*B6412, associated with 4 STs (10979, 10980, 15 768 and 20686) but with 15 nucleotide differences. However, its ST could not be determined using the NGMAST allele combination of *tbp*B1844 and *por*B6412.

### Molecular characteristics of *N. gonorrhoea* using the *por*B (510 bp) gene

**Fig. 3.**
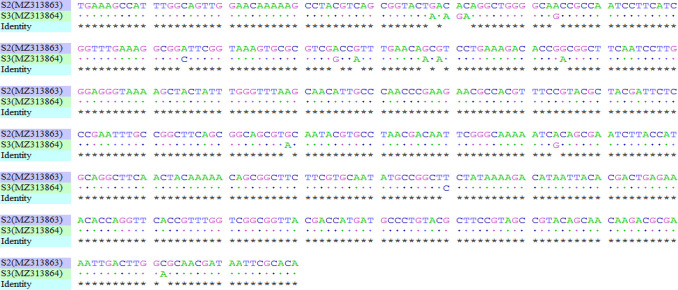
Pairwise sequence alignment of S2 (MZ313863) and S3 (MZ313864) (510 bp) shows 15 singleton variations. * indicates similarities.

A blast search on NCBI’s GenBank with S3 (*por*B) revealed 100 % identity with 25 deposited gonococcal sequences. These include WHO genome assemblies N (accession: LT591910.1) and G (accession: LT591898.1). Other 100 % identities were partial coding sequences of the gene, such as KF421819.1 (Germany), GQ289460.1 (PR China), EU530748.1 (Russia), AF090820.1 (Kenya) and AF304403.1 (USA). On the other hand, S2 (*por*B) recorded the highest identity of 99.61 % with three deposited sequences with the following accession numbers: AF200756.1 (USA), AF090810.1 (Kenya) and AF090809.1 (Kenya). The pairwise alignment of S2 (*por*B 510 bp) using default ClustalW settings on GENtle (V1.8.0) identified only two nucleotide differences with AF200756.1. S2 and S3 sequences were identified as *por*B IA alleles and assigned GenBank accession numbers MZ313863 and MZ313864, respectively. Fifteen singleton variable sites were identified between S2 (MZ313863) and S3 (MZ313864)**,** as confirmed by the pairwise alignment shown in Fig. 3.

### Molecular characteristics (haplotype network analysis)

**Fig. 4.**
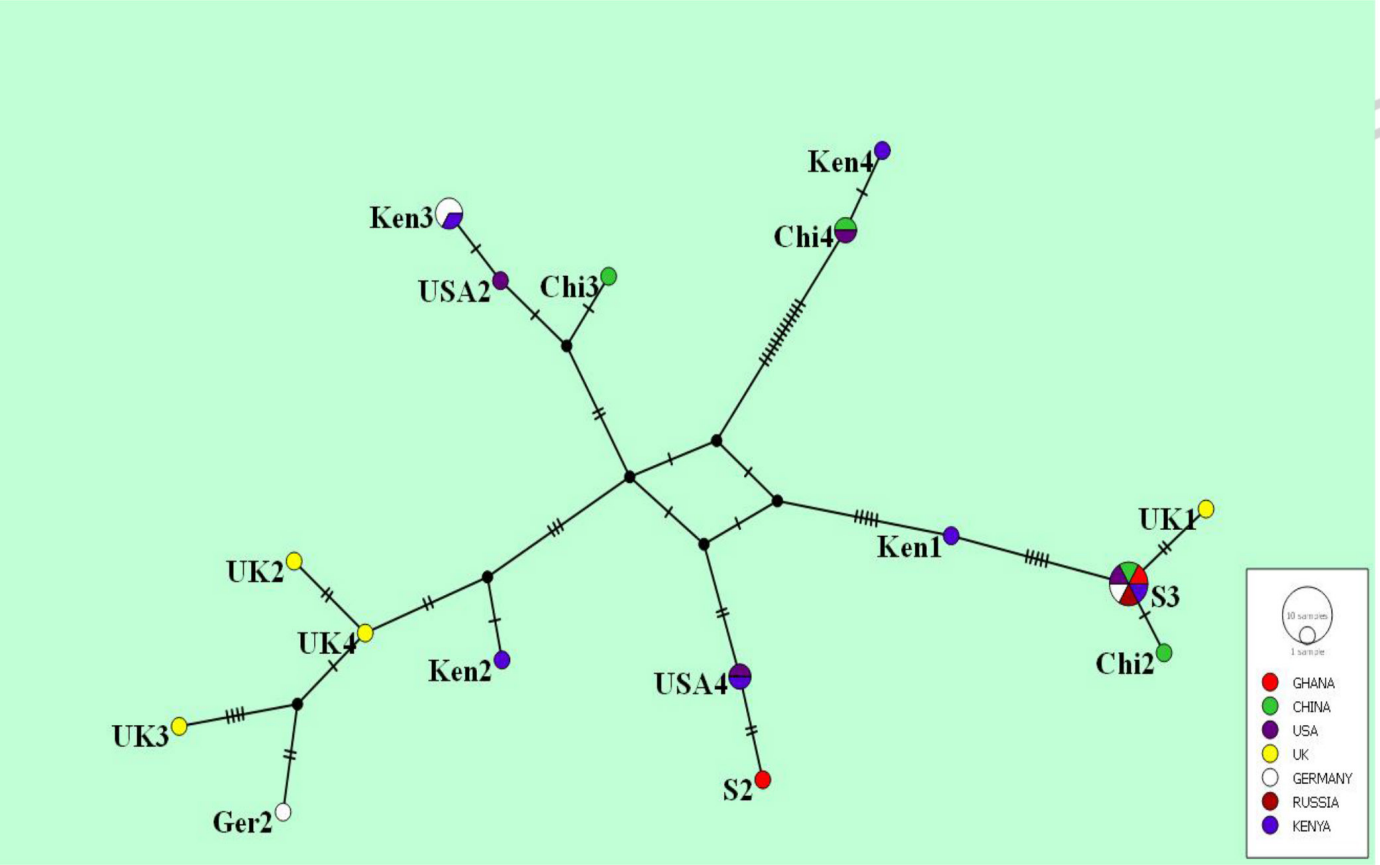
Global haplotype network of *N. gonorrhoea por*B gene (510 bp) showing S3 (MZ313864) clustering with previously reported haplotypes and S2 (MZ313863) distantly related to this cluster. Numbers in parentheses indicated on the lines represent the number of mutations between haplotypes. Coloured circles represent haplotypes previously identified in other countries, as shown in the key. Smaller black circles/dots represent missing haplotypes.

The global haplotype analysis shows the relationship between S2 (MZ313863) and S3 (MZ313864) and 23 other isolates found worldwide. S3 (MZ313864) clustered with previously reported haplotypes from PR China, the UK, Russia, the USA and Kenya, whilst S2 (MZ313863) is distantly related to the cluster, as seen in Fig. 4.

### Phylogenetic analysis of *N. gonorrhoea* using *por*B (510 bp) genes

**Fig. 5.**
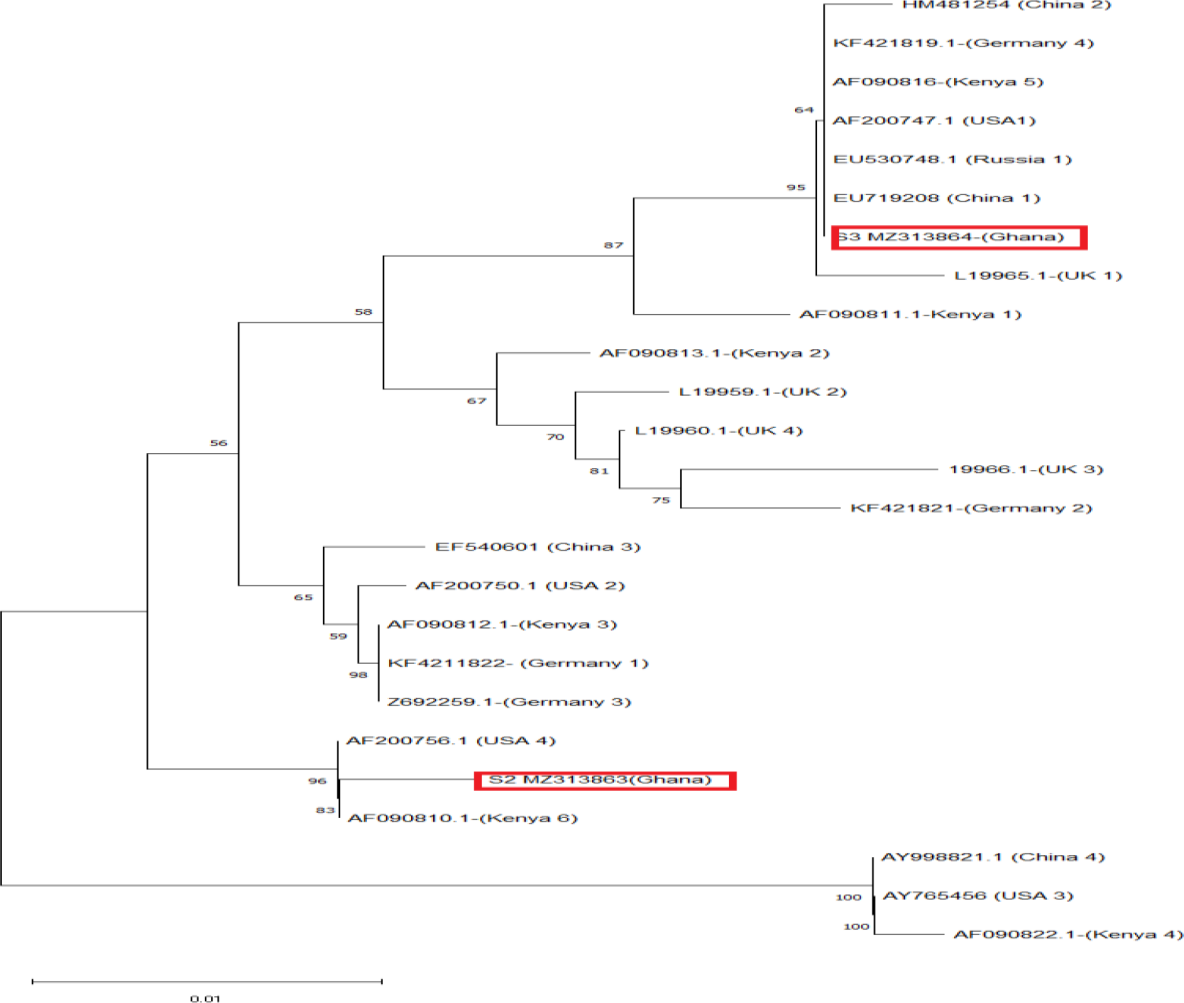
Genetic phylogram showing evolutionary relatedness of isolates of *N. gonorrhoea* (*por*B gene, 510 bp) from this study relative to 23 others found elsewhere. The percentage of replicate trees in which the associated taxa clustered together in the bootstrap test (1000 replicates) is shown next to the branches. The evolutionary distances were computed using the maximum-composite-likelihood method and are in the units of the number of base substitutions per site.

Phylogenetic analysis of the two haplotypes identified in this study with 23 other sequence deposits from GenBank based on the *por*B gene (510 bp) showed S2 (MZ313863) forming a clade with GenBank sequences from the USA (AF200756.1) and Kenya (AF090810.1). The *por*B sequence of S3 (MZ313864) formed a monophyletic clade with sequence deposits from Germany (KF421819.1), PR China (EU719208.1, HM451254.1), Russia (EU530748.1), Kenya (AF090816.1) and the USA (AF200747.1) as shown in Fig. 5.

## Discussion

Globally, frantic efforts are being made to further understand the biology, pathogenesis, AMR trajectory and determinants of *N. gonorrhoea* in the face of a ‘superbug’ status. Admittedly, this study described a very limited number of gonococcal isolates collected from an antenatal surveillance study in Tamale and routine clinical diagnosis in the selected facilities in Accra. Nonetheless, this study contributes to bridging the scanty *N. gonorrhoea* data gap in the WHO Africa region relative to other WHO regions, such as the Americas and Europe.

### Antimicrobial resistance profile of the *N. gonorrhoea* isolates

There is currently no known licensed vaccine for gonorrhoea; therefore, antimicrobial therapy has been the only resort for its management. However, gonorrhoea antimicrobial treatment is being threatened by the faster rate of AMR tendencies of *N. gonorrhoea* [5]. In Ghana, the first-line recommended gonorrhoea treatment is the WHO-recommended ceftriaxone (250–500 mg), together with the chlamydial treatment, azithromycin (1 g) [18].

The antibiotics employed for antimicrobial susceptibility testing are presently in use or were previously used in Ghana and elsewhere to treat gonorrhoea. The four antibiotics (tetracycline, penicillin, erythromycin and tagomycin) resisted by all the isolates in this study, indicating 100 % multi-drug resistance, were previously reported in Ghana [17, 20], Iran [28] and Ethiopia [29]. This indicates that these antibiotics are still resisted by circulating *N. gonorrhoea* years after their withdrawal [5] as therapeutic agents. The recorded resistance (50 %) and intermediate susceptibility (50 %) to ciprofloxacin have also been recorded previously [17, 20], which supports its replacement as a first-line treatment regimen by the Ghana Health Service in 2018. Nonetheless, despite the current recommended first-line dual-treatment regimen of ceftriaxone and azithromycin, ciprofloxacin is still used as first-line regimen in some parts of Ghana for initial treatment of gonorrhoea. The application of ciprofloxacin in these areas suggests that the circulating gonococcal strains may still be susceptible to the antibiotic. The ability of *N. gonorrhoea* to resist fluoroquinolones such as ciprofloxacin is provided by *gyrA* mutations, reducing its binding affinity [5, 30]. Resistance and reduced susceptibility to azithromycin by *N. gonorrhoea*, as observed in this study, was first reported in Argentina in 2001 [31] and became a global concern by 2010 [32–34]. Azithromycin resistance has previously been reported in Ghana [15]. However, it is still recommended in the current globally recommended dual-treatment regimen of gonorrhoea with ceftriaxone [35]. Transmutations in either the *mtrR*, *erm* or *mef* genes, which encode the efflux pump that results in the overexpression of the efflux pump systems in *N. gonorrhoea*, have been linked to reduced sensitivity to azithromycin [36–38].

The treatment efficacy of the extended-spectrum cephalosporins (ESCs), including ceftriaxone, has been widely reported, hence its current recommendation for the treatment of gonorrhoea [39–41]. However, the treatment failures and reduced susceptibility recorded in this study had been reported earlier [42, 43] and recently in Ghana [17]. The primary resistance factor for ESCs has been linked to the alleles of mutant mosaic penicillin-binding protein two (PBP2) and a non-mosaic PBP IX harbouring a P551L substitution [44]. Reported resistance to the last-resort ESC antibiotics is a threat to treatable gonorrhoea. The Tamale isolate S2 (MZ313863) showed the same antibiotic resistance profile for the tested antibiotics as the Accra isolate S3 (MZ313864). The other Accra isolates (S1 and S4) showed different resistance profiles, with S4 recording resistance to all the tested antibiotics. The differences in the antibiotic resistance profiles recorded by all three Accra isolates showcases the heterogeneity of the circulating strains in Ghana even though further studies is required to confirm this observation. Addtionally, the high MAR index (>0.2) observed in all the antibiotics in this study suggests extensive uncontrolled use of these antibiotics and/or the observed ‘superbug’ tendencies of the gonococcus. The outcome of this study’s antibiotic profile supports widespread apprehension about *N. gonorrhoea’*s ability to develop into a ‘superbug’ despite the small number of isolates studied. This is particularly important for developing countries, where syndromic management is recommended for treating gonorrhoea and other STIs.

### Molecular characteristics of *N. gonorrhoea* isolates

The NG-MAST sequence typing revealed two genotypes. Sequence type 211 (ST211), identified with isolate S3 (MZ313864), has previously been reported in the UK, Germany and New Zealand, and has been associated with decreased resistance to ciprofloxacin [45, 46]. Locus 1844 (*tbp*B gene), identified with S2 (MZ313863), was found with only three NG-MAST STs, i.e. ST10251, ST19711 and ST19719, indicating that it may not be a widespread allele. Its *por*B sequence query returned the closest allele, 6412, associated with 4 STs (10 979, 10 980, 15 768 and 20 686) but with 15 nucleotide differences. Nonetheless, neither allele type could yield a known ST. Similarly, the extended version of S2 *por*B (510 bp) (MZ313863) was 99.61 % similar to other sequence deposits such as AF200756.1, with only two nucleotide differences in the NCBI’s GenBank. This indicates that not all sequence deposits in the NG-MAST database have been deposited in GenBank, and vice versa. It also suggests that S2 *por*B (MZ313863) is a unique variant of gonococcal *por*B identified in Tamale. Following these observations, it will be prudent for all sequence deposits in the NG-MAST database to be deposited in GenBank, and vice versa, for easy reference. This situation calls for the continued integration of all currently available gonococcal typing schemes, as is being endeavoured on the pubMLST platform. Additionally, the International Pathogenic Neisseria Conference (IPNC) and the *Neisseria gonorrhoea* Research Society (NgoRS) should continue to serve as platforms for the scientific community for further consolidation of all gonococcal studies onto one platform. Neither the identified NG-MAST ST211, the *tbp*B allele type 1844 nor the *por*B allele type 6412 found in this study had previously been reported in Ghana, indicating the heterogenicity of *N. gonorrhoea* isolates in the country.

S2 (MZ313863) and S3 (MZ313864), identified as *por*B IA, are associated with disseminated gonorrhoea [47], which may cause meningitis, endocarditis, arthritis and septicaemia, among other deleterious complications [48].

Despite the two sequencing attempts, the unreadability of the sequence chromatograms of S1 and S4 isolates is not readily known, even though the crude DNA extraction process and/or Inqaba Biotec LTD sequencing processes cannot be overlooked. Notwithstanding the similarity of the antibiotic resistance profiles recorded in this study, the significant genetic variation between S2 (MZ313863) and S3 (MZ313864) points to a wide genetic diversity of gonococcal strains in Ghana. The origin of the observed intercontinental genealogical relatedness of haplotype S3 (MZ313864) is not readily known. While the *por*B sequence of S2 (MZ313863) showed (99.61 % similar to three others) perhaps a novel strain, more gonococcal samples need to be studied in the Tamale Metropolis, where it was isolated to confirm and ascertain its spread. This study corroborates an earlier study in southern Ghana [15], where varied strains of *N. gonorrhoea* were identified.

The 15 singleton variable sites observed between the two isolates is evident in their distanced clustering in the global haplotype analysis and their formation of different monophyletic groups. Relative to the other isolates found globally, it suggests that either these strains were transmitted through travel or might have developed independently as a result of undergoing similar stress. Evolutionary history also indicated that S2 (MZ313863) shared a common ancestor before at least six-ancestry evolution that resulted in the clade involving S3 (MZ313864). To emphasize this, continuous assessment of local circulating strains of *N. gonorrhoea* in Ghana, especially in the northern part of the country, would enhance the knowledge of circulating gonococci strains. This information would help inform appropriate local therapeutic regimens while contributing to global surveillance.

## Conclusion

This study recorded two strains of *N. gonorrhoea* from four molecularly confirmed *N. gonorrhoea* isolates from Accra and Tamale. Multidrug resistance was observed, with all four *N. gonorrhoea* isolates, showing resistance against at least four antibiotics. One of the Accra isolates (S4) was resistant to all seven antibiotics. Molecular typing of the Tamale isolate S2 (MZ313863) suggested a possible new strain. There is therefore the need for continued AMR and molecular surveillance, and expedited action to develop a gonorrhoea vaccine before there are widespread deaths due to untreatable gonorrhoea.
